# Human myelomeningocele risk and ultra-rare deleterious variants in genes associated with cilium, WNT-signaling, ECM, cytoskeleton and cell migration

**DOI:** 10.1038/s41598-021-83058-7

**Published:** 2021-02-11

**Authors:** K. S. Au, L. Hebert, P. Hillman, C. Baker, M. R. Brown, D.-K. Kim, K. Soldano, M. Garrett, A. Ashley-Koch, S. Lee, J. Gleeson, J. E. Hixson, A. C. Morrison, H. Northrup

**Affiliations:** 1 Division of Medical Genetics, Department of Pediatrics, McGovern Medical School, University of Texas Health Science Center at Houston, Houston, TX 77030 USA; 2 Department of Epidemiology, Human Genetics and Environmental Sciences, School of Public Health, University of Texas Health Science Center At Houston, Houston, TX 77030 USA; 3 Department of Medicine, Duke University Medical Center, Durham, NC 27701 USA; 4 Department of Neurosciences and Pediatrics, University of California-San Diego, La Jolla, CA 92093 USA; 5 Rady Children’s Institute for Genomic Medicine, San Diego, CA 92025 USA; 6 Present Address: Munroe-Meyer Institute, University of Nebraska Medical Center, Omaha, NE 68198 USA

**Keywords:** Developmental biology, Genetics, Neuroscience, Diseases, Molecular medicine, Neurology, Risk factors

## Abstract

Myelomeningocele (MMC) affects one in 1000 newborns annually worldwide and each surviving child faces tremendous lifetime medical and caregiving burdens. Both genetic and environmental factors contribute to disease risk but the mechanism is unclear. This study examined 506 MMC subjects for ultra-rare deleterious variants (URDVs, absent in gnomAD v2.1.1 controls that have Combined Annotation Dependent Depletion score ≥ 20) in candidate genes either known to cause abnormal neural tube closure in animals or previously associated with human MMC in the current study cohort. Approximately 70% of the study subjects carried one to nine URDVs among 302 candidate genes. Half of the study subjects carried heterozygous URDVs in multiple genes involved in the structure and/or function of cilium, cytoskeleton, extracellular matrix, WNT signaling, and/or cell migration. Another 20% of the study subjects carried heterozygous URDVs in candidate genes associated with gene transcription regulation, folate metabolism, or glucose metabolism. Presence of URDVs in the candidate genes involving these biological function groups may elevate the risk of developing myelomeningocele in the study cohort.

## Introduction

**Figure 1:**
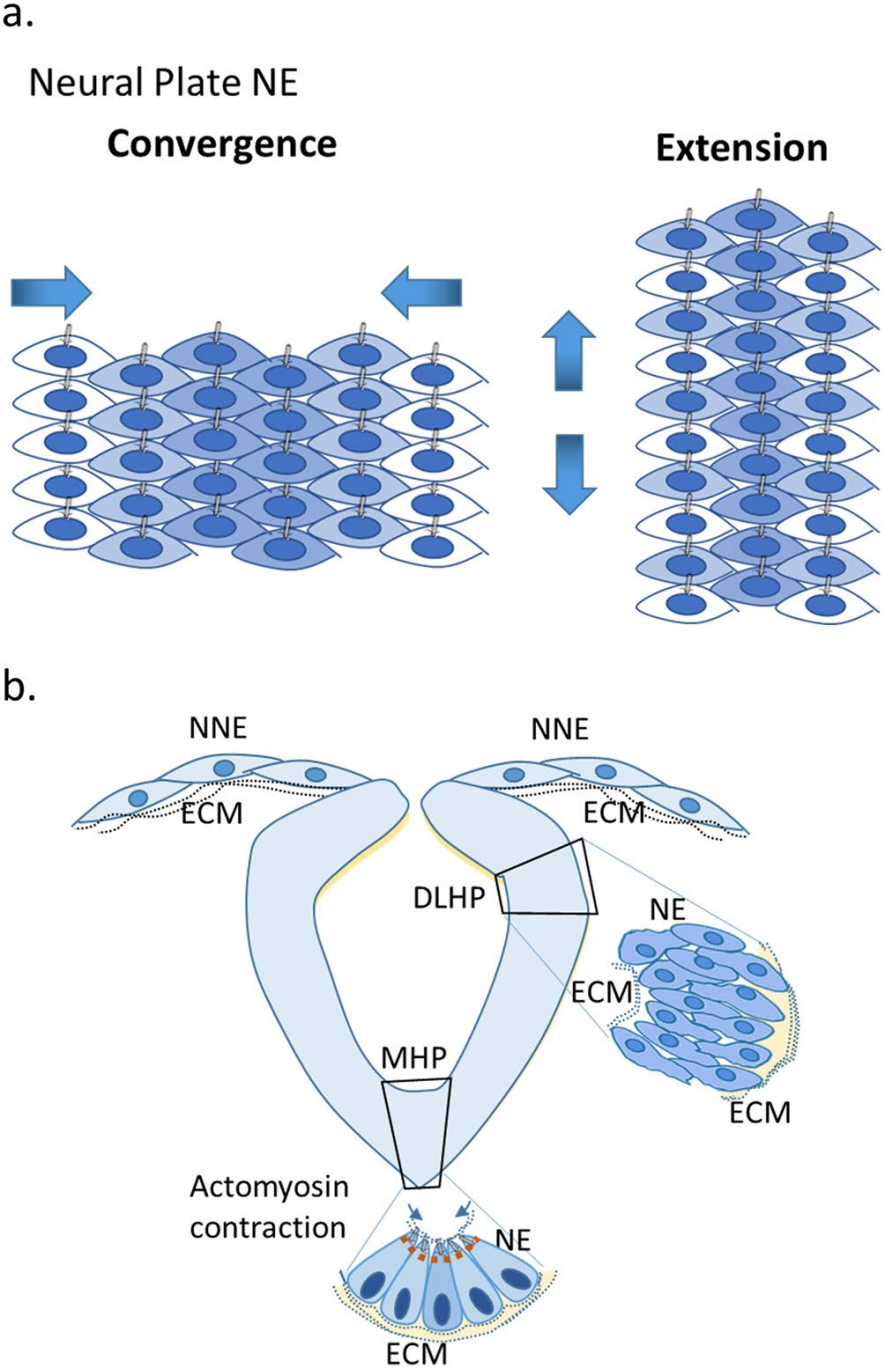
Neural tube formation involves a series of biological processes including mechanochemical signal sensing from the environment to orchestrate cell proliferation, cytoskeleton remodeling, and migration of neural and non-neural ectodermal cells. (**a**) Neural plate cells undergo convergent-extension to facilitate posterior-anterior elongation of the neural tube. Neuroectodermal cells (NE) migrate to the midline in response to extracellular signals received by cilia in NE cells to establish and maintain polarity. (**b**) Midline NE cells undergo apical constriction via a biological process involving actomyosin cytoskeleton remodeling to form the midline hinge point (MHP). NE cell and non-neuroectodermal (NNE) cells continue proliferation and migration during the neural tube closing process upon sensing signals in the extracellular matrix (ECM). NE cells at the dorsolateral hinge point (DLHP) undergo proliferation, migration dorsally, and reshaping to form the DLHP bringing the NE/NNE edges at the dorsal midline together. Advancing migration and proliferation of NNE and mesenchymal cells facilitate ectodermal layer extension when dorsal midline cells’ protrusions meet and join at the closing point. The process also brings NE cells together to establish cell junctions and close the NT.

Successful neural tube formation relies on a series of orchestrated biological processes to facilitate convergent extension of neural ectodermal cells (NE) on the neural plate. NE cells at the midline and the dorsolateral regions form the medial hinge point (MHP) and the dorsolateral hinge point (DLHP) through mechanisms including apical construction and localized cell proliferation, and proliferation of non-neural ectodermal cells (NNE) that facilitate closing of the neural tube^1^ (Fig. 1). These biological processes involve sensing signals in the extracellular matrix by signal receptors on the cell surface and sub-organelles (e.g. mechanochemical sensors on primary cilia) which subsequently trigger remodeling of cytoskeletal structures and orchestrate directional migration of NE and NNE cells^2^. The neural tube can fail to close due to impaired convergent extension, MHP or DLHP formation, structural integrity of NE, or midline fusion of NE or NNE^3^. Maintaining NNE integrity and controlling epithelial-to-mesenchymal-transition of neural crest cells^4^, and balancing proliferation of hindgut cells^5^ (of endodermal origin) may also play important roles in the development of neural tube. Synchrony of all these processes together transform the neural plate into neural folds that meet and merge to form the neural tube. Factors interrupting the structure and/or function and spatial temporal integrity of these synchronous processes could result in failure of neural tube closure^1,3^. More than 400 genes have been identified in animal models that are required to maintain normal neural tube closure^6–8^. Many of the neural tube defect (NTD) animal model genes constitute structural and/or functional components of cilium, extracellular matrix, the WNT signaling pathway, actomyosin cytoskeleton remodeling and cell migration^3,9^.

An increasing number of heterozygous deleterious genetic variants in genes regulating planar cell polarity (PCP) have been revealed in human NTD cases and suggest that multi-genic heterozygous deleterious variants may contribute to NTD development^10–13^ . Knowledge is lacking regarding the extent of deleterious variants in genes involving various biological processes contributing to human myelomeningocele (MMC). It is possible that defective genes not known to interact with PCP genes in combination with defective PCP genes together can confer risk of MMC. Genes of interest for this study included variants of 551 candidate genes (537 mouse genes and 14 human NTD associated genes) previously shown to cause NTDs in mouse embryos or previously associated with human NTD (see Supplementary Table 1). In a complex trait study, the effect size of genetic variants has been shown to be inversely related to the frequency of the alternate allele of variants^14^, and new and private variants could constitute an important reservoir of disease risk alleles^15^. Single nucleotide variants (SNVs) in 551 candidate genes that were absent in non-Finnish European (NFE) or Ad-mixed American (AMR) in gnomAD v2.1.1 (controls) and which have top 1% deleteriousness (Combined Annotation Dependent Depletion phred score, C-score ≥ 20)^16^ were catalogued and defined as ultra-rare deleterious variants (URDVs) for analysis in the study. The majority of the homozygous defective mouse genes caused open neural tubes in the cranial regions whereas heterozygous mice had normal neural tube development^9^. Interestingly, some mice with a subset of these genes in the digenic heterozygote condition developed spina bifida, suggesting that oligogenic heterozygosity is a possible mechanism for spina bifida development^17^. Recent human spina bifida studies revealed cases carrying only heterozygous damaging variants of PCP genes and examples of subjects with digenic heterozygote rare variants in some PCP genes were observed^13^. In addition, some cases had damaging variants in two PCP genes each inherited from one of the parents^12^. Oligogenic inheritance is a demonstrated disease mechanism in many human diseases including the birth defect with holoprosencephaly^18^. Representation of oligogenic heterozygous URDVs in two or more NTD candidate genes found in MMC subjects in the study will be reported. Ontology analysis suggested URDV-containing NTD candidate genes were enriched for the same ontology group or in multiple categories including cilium, cytoskeleton, extracellular matrix, WNT signaling, and cell migration. The potential contribution of the observed results to MMC development in humans will be discussed.

## Results

### Global landscape of single nucleotide variants of MMC exomes

Three European (EA) and two Mexican (MA) MMC subjects had variant counts less than 80% of the median variant counts of the study subjects and were removed from further analysis, leaving 254 EA and 252 MA MMC exomes. A total of 597,401 high confidence SNVs that passed filtering steps described in the Materials and Method section were selected for further analysis. Using dbNSFP v4.0 to annotate high quality SNVs, 112,369 variants predicted to be functionally significant in 16,135 genes were selected for further analysis that included 109,727 missense, 1814 stop_gained, 84 stop_lost and 771 splice site variants. Non-coding SNVs and synonymous SNVs were not analyzed in this study.

Using AMR and NFE datasets from gnomAD v2.1.1 (controls) as references, approximately 15.1% and 13.2% of the SNVs in EA MMC and MA MMC respectively were ultra-rare variants (URVs) with alternate allele frequency (aAF) in the gnomAD v2.1.1 (controls) equal to zero or with an absence of the known variant. URVs found only in MMC subjects may constitute an important enriched pool of risk alleles for further analysis^15^. An average of 40 URVs per subject were identified. Over 98% of URVs occurred only once. Approximately 7400 URVs had C-score ≥ 20 in each ethnic group (see Supplementary Table 2). These variants were defined as ultra-rare functionally deleterious SNVs (URDVs) and used for further analysis. Each study subject had approximately 29 URDVs with range between 9 and 90.

Eighty-eight subjects were re-sequenced using the Illumina NGS platforms. Of the 30,789 SNVs in 33 EA and 37,924 SNVs in 55 MA identified from the Ion Proton platform in this study, over 98.5% were also identified by the Illumina NGS platforms.

#### Landscape of URVs in NTD candidate genes in human MMC exomes

There are several hundred genes known to cause NTDs in mice under monogenic and oligogenic conditions^3,9^. We sought insight through examining SNVs in 551 NTD candidate genes (see Supplementary Table 1) known to cause abnormal neural tube closure in animal models (537 genes), and 14 genes associated with human MMC in our study cohort. A total of 2134 SNVs in 440 genes were identified from EA MMC exomes and 2496 SNV in 447 genes were identified from MA MMC exomes.

#### Ultra-rare functional deleterious SNVs (URDVs) in candidate genes

**Figure 2:**
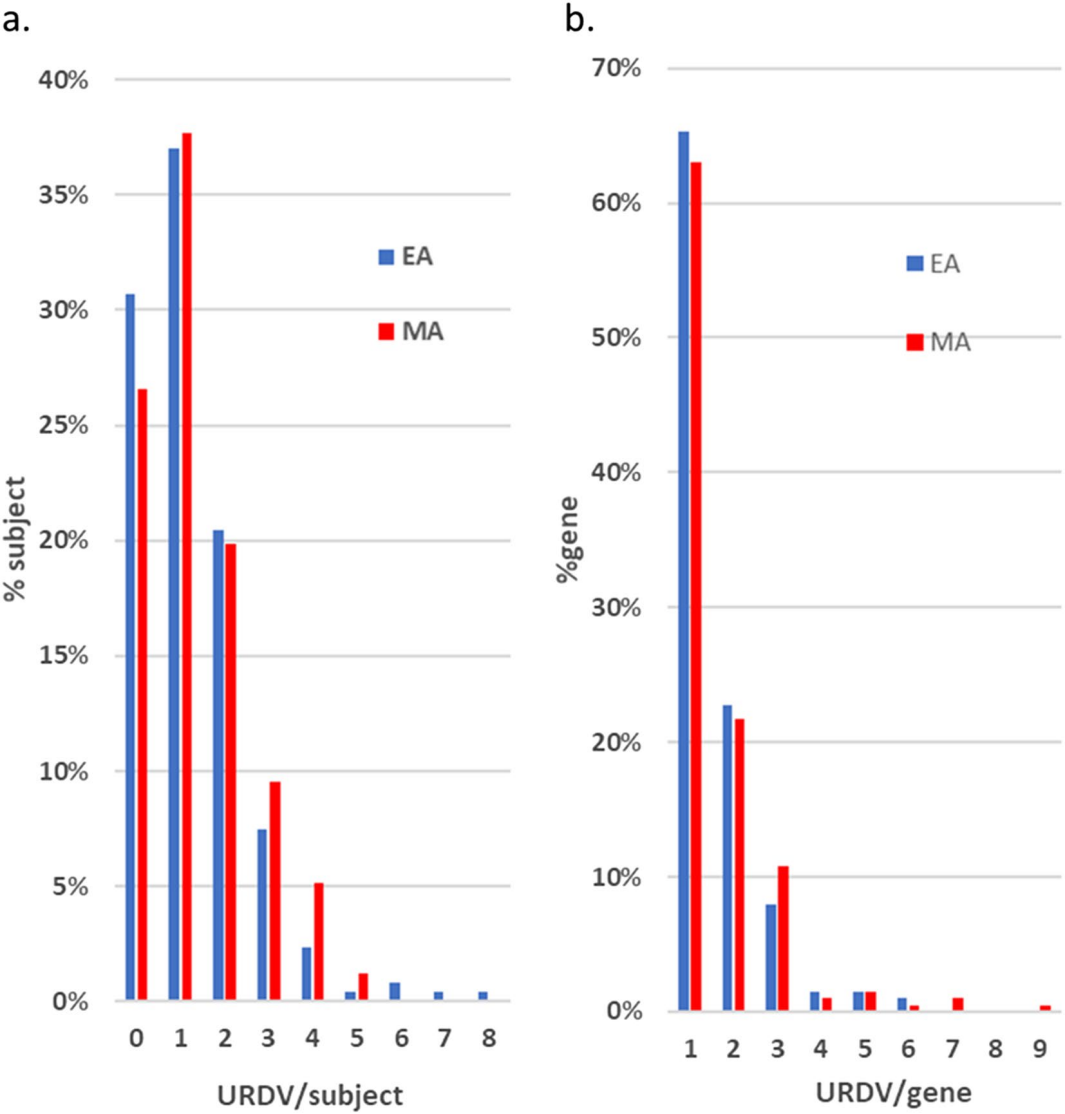
Distributions of URDVs Identified by WES. (**a**) Chart shows count of URDVs in NTD candidate gene(s) identified per exome of MM subjects varied from 0 to 8. In this study, the number of EA MMC subjects is 254 and the number of MA MMC subjects is 252. Approximately 25–30% subjects had no URDVs in NTD candidate genes, around 35% had one URDV, and the remaining had more than one. (**b**) Shows counts of URDVs discovered per NTD candidate gene ranged from one to nine with nearly 65% genes containing only one. Total number of URDV-containing NTD candidate genes found in EA and MA MMC subjects are 202 and 203 respectively.

Many studies have suggested the impact of variants is inversely proportionate to the alternate allele frequencies (aAF)^14^. Using aAF of variants in NFE/AMR gnomAD (controls) exomes as a filter, all 506 MMC subjects carried 31–69 rare deleterious variants (RDVs, aAF ≤ 0.01) in NTD candidate genes whereas the number of URDVs per subject varied between zero and eight (see Fig. 2 & Supplementary Table 3). With increased findings of novel and very rare variants in PCP genes associated with human NTDs in many previous studies^3,19^, we chose to focus on the novel URDVs, which are more likely to have higher effect size, for subsequent analyses.

Among 254 EA MMC subjects there were 307 URDVs in 202 genes. On average, each URDV-containing gene in EA MMC group had 1.52 URDVs. The range of URDVs per gene found in EA MMC was zero to six. Among 252 MA MMC subjects, there were 331 URDVs found in 203 genes. An average of 1.63 URDVs in a URDV-containing NTD candidate gene was identified in MA MMC group. The range of URDVs per gene found in MA MMC subjects was zero to nine. Over 97% of URDVs had an allele count (AC) of one and a few had two to three. The majority (~ 95%) of URDVs were missense changes while the remaining ~ 3.4% were stop_gained or splice site changes (1.3% and 2.4% in EA and MA groups respectively). Around 70% of MMC subjects in both ethnic groups had at least one URDV in an NTD candidate gene, with the highest URDV counts being eight and five in EA MMC and MA MMC subjects, respectively (Fig. 2). There were 78 EA MMC subjects and 67 MA MMC subjects who had no URDVs in NTD candidate genes.

URDV-containing NTD candidate genes were either common to both ethnicities or found uniquely in EA MMC and MA MMC exomes. There were 99 genes unique to EA MMC, 100 genes unique to MA MMC and 103 genes were common to both ethnic groups. Examining EA MMC and MA MMC respectively, most (66.7% and 63.4%) of the genes had one URDVs; 21.3% and 22.1% had two; 10.9% and 7.4% had three and the remaining few had four to nine URDVs. For the entire study cohort, 636 URDVs with 603 missense, 21 stop_gained and 12 splice site changes in 302 NTD candidate genes were discovered (Supplementary Table 1). These URDVs were manually verified using the online Integrative Genomics Viewer (IGV) Web App (https://igv.org/app). Seven subjects carried two heterozygous URDVs in their candidate genes (Supplementary Table 3). Visual inspection using IGV revealed the two heterozygous URDVs were in cis positions in the candidate genes (i.e. *FREM2, FOLR2, RPGRIP1L, BMP2*, and *CGN*) of five subjects respectively. One subject (i.e. C35-874, Supplementary Table 3) had two heterozygous URDVs in trans position in the *KIF7* gene. The remaining subject (i.e. F57-264, Supplementary Table 3) carried two URDVs 1400 bp apart in two different exons of *PNPLA6* therefore, we were unable to determine whether the URDVs were in cis or in trans.

**Figure 3:**
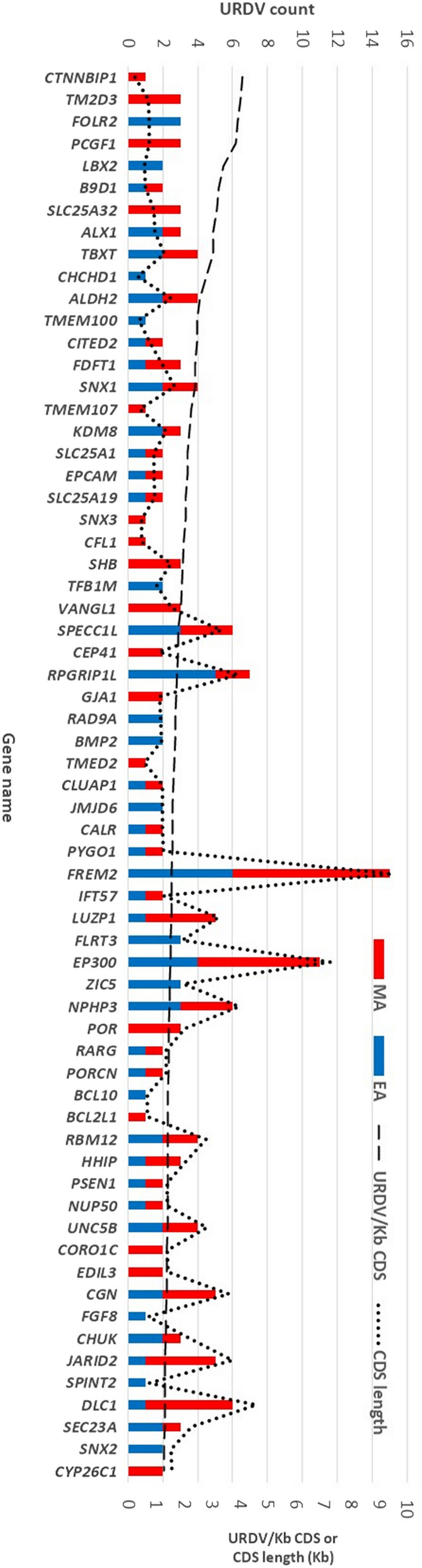
URDV density per Kb coding sequence of NTD candidate genes. The majority of 302 NTD candidate genes show URDV/KbCDS density less than one. The x-axis showed the gene names ordered by the URDV/Kb CDS from highest (4.1 for *CTNNBP1*) to lowest (1.28, *CYP26C1*). Dashed line (- - - -) showed the URDV/Kb CDS ratio of the gene and dotted line (…….) showed the length of CDS in Kb. Count of URDV in EA showed in blue bar and MA showed in red bar. Approximately half of the 64 genes had URDVs found in both EA and MA. One-third of the 64 genes have ≥ 2 URDVs/Kbp CDS.

The number of URDVs identified was not directly proportionate to the length of coding sequence (CDS) of genes in the two subject groups (Supplementary Table 3). Genes with longer transcript length (CDS length > 10 Kb) had a higher number of URDVs as expected (e.g. *FREM2, HSPG2, APOB, DYNC2H1* and *HTT*) although their URDVs/Kb CDS was less than one. Overall, 96 genes had URDV densities of over one per Kb of CDS and the 64 genes had ≥ 1.25 URDVs/Kb CDS (Fig. 3, Supplementary Table 3). These 96 genes harboring 272 URDVs accounted for over 40% of the total 636 URDVs identified in 302 candidate genes. The median size of the CDS for the 96 genes was 1.39 Kb.

Nine genes in MA MMC subjects had a higher number of URDVs, ranging from four to nine, with the most in *FREM2*. In EA MMC subjects, eight genes had four to six URDVs with six in *APOB* and in *FREM2*. Three genes (*EP300, HSPG2,* and *FREM2*) found in both EA and MA MMC subjects had more than ten URDVs with *FREM2* and *EP300* had an URDV density around 1.5.

#### Ontology Enrichment of NTD candidate genes with URDVs in MMC exomes

**Figure 4:**
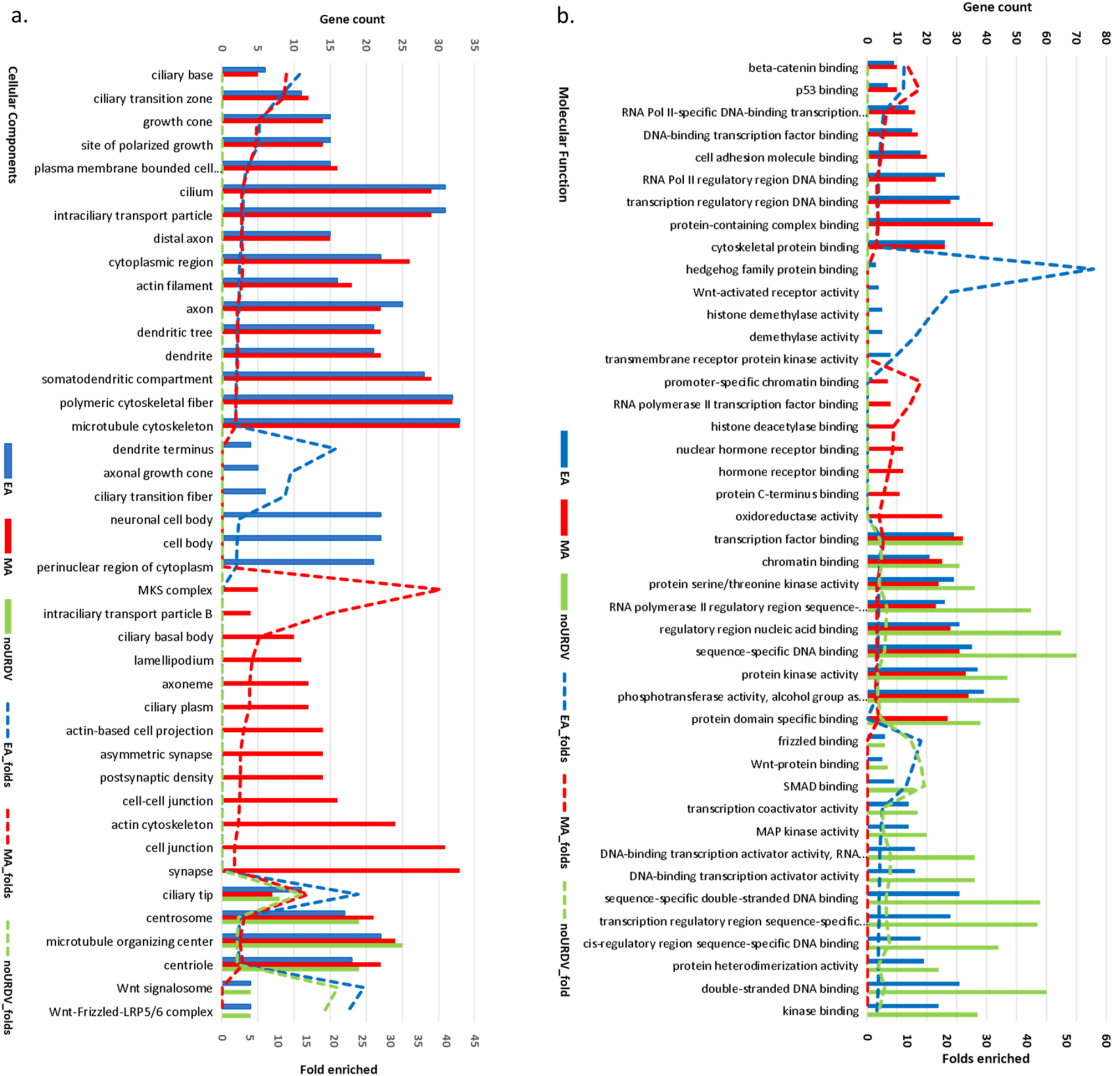

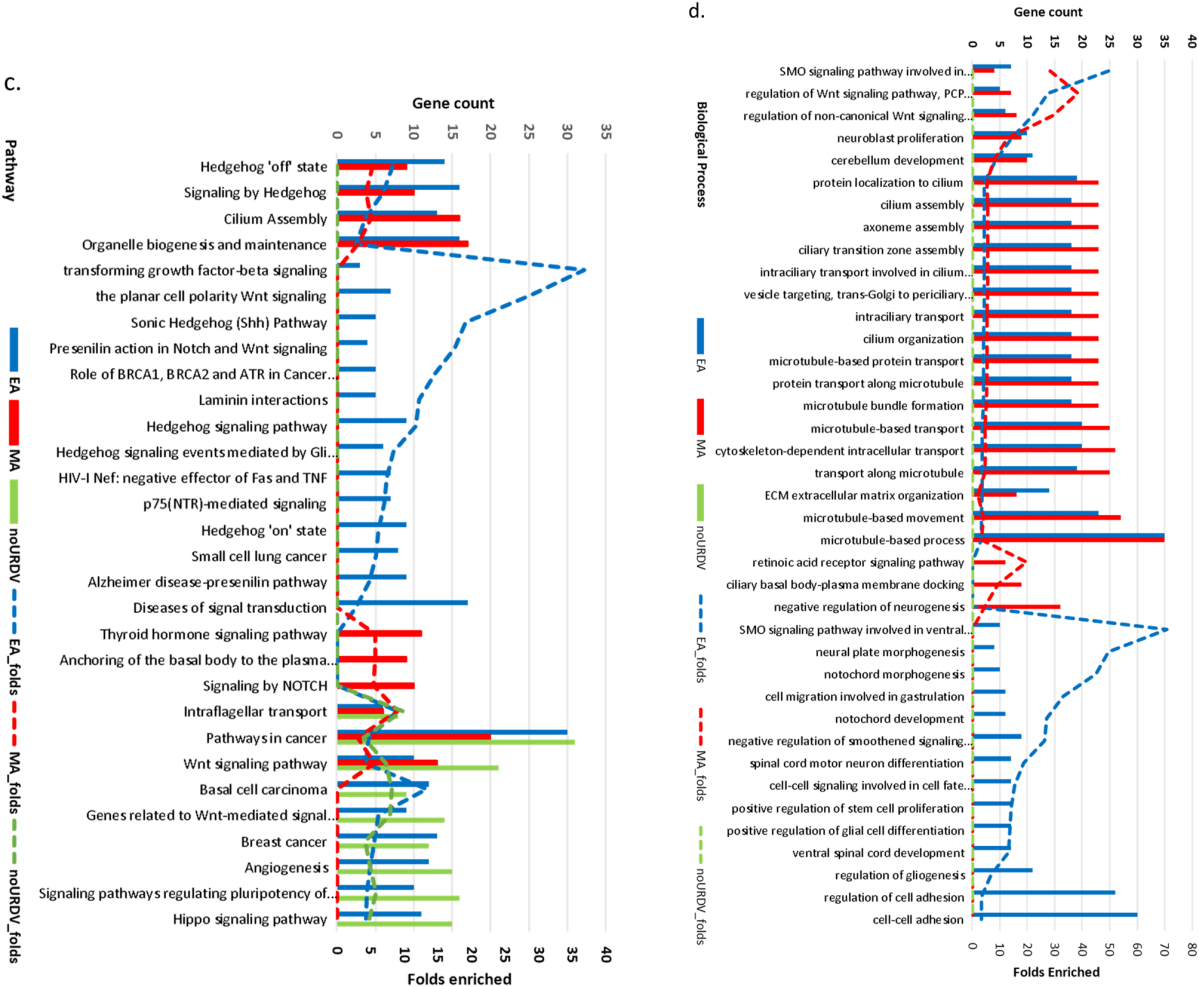
Results of ontology and pathway enrichment analysis of candidate genes with and without URDVs identified. Enrichment analysis of (**a**) cellular components, (**b**) molecular functions, (**c**) pathways, and (**d**) biological processes, were performed using online tools ToppFun and ToppCluster. Count of URDV-containing genes in ontology class for EA (blue) and MA (red) MMC subjects and genes without URDVs (green) were shown with bar charts. Enrichment folds were represented with dashed lines for URDV-containing genes in EA (blue), URDV-containing genes in MA (red) and genes without URDVs (green). Enrichment comparisons passed Bonferroni correction. Detail descriptions of ontology, gene names, fold enrichment, and Bonferroni corrected *P* values can be found in Supplementary Table 4.

Among the 551 NTD candidate genes, enrichment of components in cilium, cytoskeleton, extracellular matrix and cell migration/adhesion, and WNT signaling were suggested (Supplementary Table 4). Using the ToppCluster^20^ online program, genes between EA and MA groups consisting of URDVs were compared to the group of genes lacking URDVs using Bonferroni correction for multiple testing. Analysis results showed genes in EA and MA groups had higher folds of enrichment in ontology classes than those of the overall 551 candidate genes. However, there was very distinct overrepresentation of subclasses of ontology for URDV-containing candidate genes in the subjects and these subclasses were absent among the candidate genes without URDVs (Fig. 4 and Supplementary Table 4). Enrichment of cellular component genes containing URDVs was seen mostly among ciliary components and intraciliary transport, actin filament, and microtubule cytoskeleton organization in both ethnic groups (Fig. 4a, and Supplementary Table 4). URDV-containing genes in EA MMC subjects were associated with components found in neuronal cell body, perinuclear region of cytoplasm, ciliary transition fiber, and WNT/SHH signaling. URDV-containing genes in MA MMC subjects were associated with components of ciliary basal body actin-based cytoskeleton and cell projection and cell–cell junctions. Differences in cellular component genes with URDV between EA and MA subjects were observed.

Molecular function analysis showed enrichment of URDV-containing candidate genes with molecular functions in transcription modulation, beta-catenin binding, cell adhesion, and cytoskeleton binding in both EA and MA MMC exomes (Fig. 4b, and Supplementary Table 4). EA MMC cases had enriched candidate genes related to hedgehog family protein and WNT signaling molecule binding. MA MMC cases had enriched candidate genes involved in DNA binding with transcription factors and nuclear hormone receptors. Together, nearly half of the MMC subjects in the study cohort had one or more URDVs in the candidate genes belonging to the ontological groups associated with ciliary structures and functions.

Both EA and MA MMC exomes consistently had URDV-containing genes disproportionately represented in the Hedgehog/WNT signaling and cancer signaling pathways (Fig. 4c, and Supplementary Table 4). Of note, EA MMC exomes uniquely enriched with URDVs in genes from WNT/PCP, SHH and TGFβ signaling pathways. MA MMC exomes were uniquely enriched with URDVs in genes related to thyroid hormone signaling, NOTCH signaling, and anchoring ciliary basal body.

Most of the NTD candidate genes involve biological processes of embryo development of organs particularly for the central nervous system and other organ systems (Supplementary Table 4). Ontology terms for biological processes are also consistent with those described in the cellular components and molecular functions sections (Fig. 4d).

**Figure 5:**
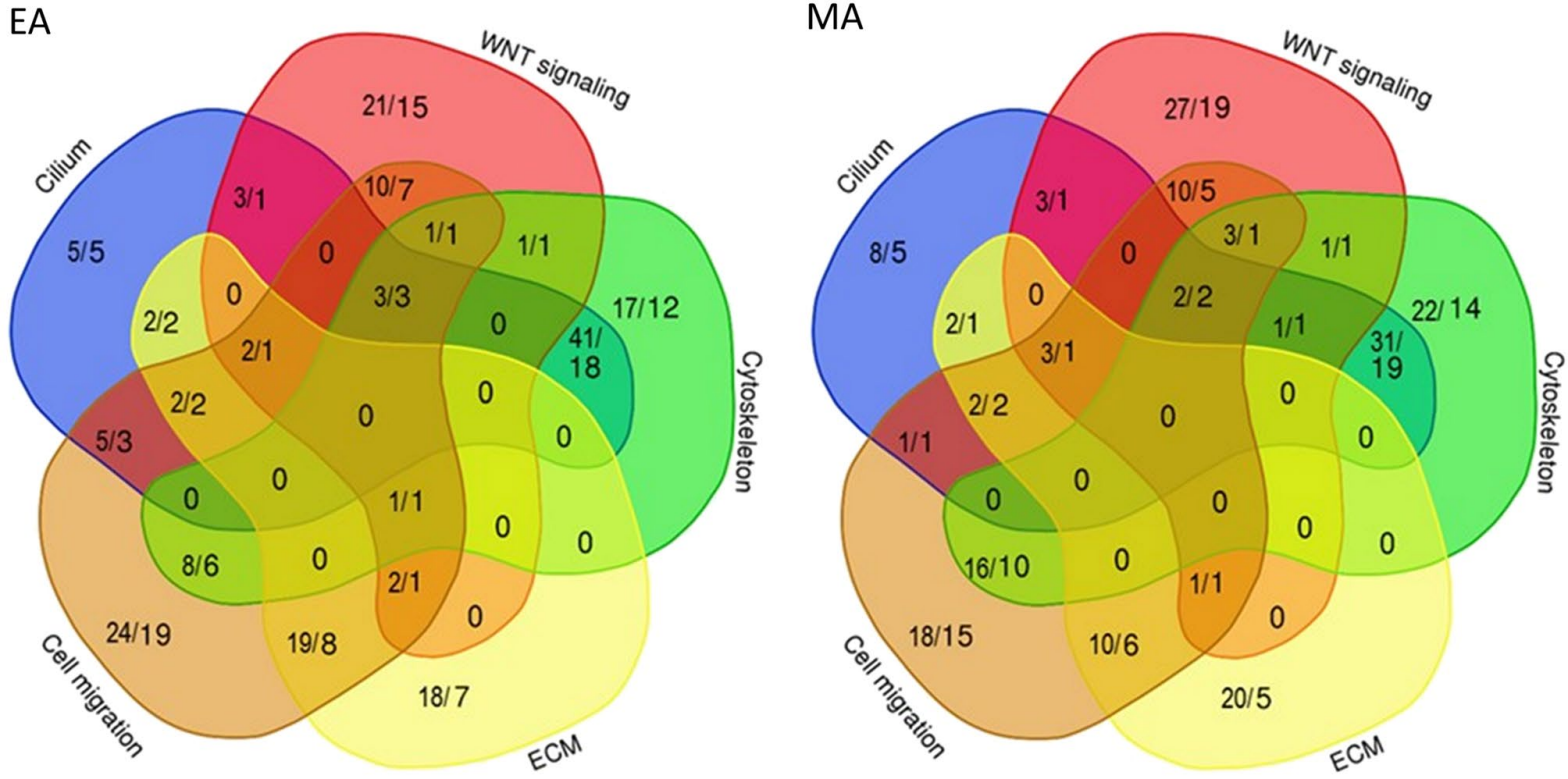
URDV containing candidate genes involved in multiple ontology groups. The Venn diagrams showed the relation and distribution of UTDV count and NTD candidate genes count to five ontology groups for EA (**a**) and MA (**b**). The number of URDVs and number of genes classified to cilium, WNT signaling, cytoskeleton, extracellular matrix (ECM) and cell migration are shown as fraction (count of URDV/count of gene). Genes classified to multiple categories were shown in the overlapped subset compartments between categories. Details on genes within each segment can be found in Supplementary Table 5.

Ciliary structure and functions are closely associated with GO terms for cytoskeleton/microtubules, extracellular matrix, WNT signaling, and migration of cells and cellular components. To further dissect the properties of URDV-containing NTD candidate genes and to examine the URDV distribution in MMC exomes, we performed analysis of genes assigned to these five GO terms (Supplementary Table 5). Candidate genes in each of these ontological groups consisted a range of 12–23.2% of the total URDVs (Table 1). Together, nearly half of the MMC subjects in the study cohort have one or more URDVs in the candidate genes belonging to the five ontological groups. There were 113 and 110 NTD candidate genes with URDVs respectively in the EA and MA subject groups that were associated with functions for cilium, ECM, cytoskeleton and WNT signaling and cell migration. Of these genes, some belong in only one GO term and many in two to four GO terms with cross-interacting roles (Fig. 5, and Supplementary Table 6). Of 364 subjects in the study cohort who bore at least one URDV in an NTD candidate gene, nearly 75% had URDVs affecting genes associated with cilium, WNT signaling, ECM, cytoskeleton and cell migration remodeling suggesting they may confer increased genetic risk to MMC development. The remaining 25% of MMC subjects had URDV-containing NTD candidate genes that were associated with other gene functions such as transcription modulation, folate one carbon metabolism network and glucose oxidative stress^21^ not related to the ontologies examined in this study.

**Table 1:** Distribution of URDVs and genes in five biological ontology groups among MMC subjects.

Categories	EA				MA			
	#URDV	#gene	#URDV/ gene	#subjects	#URDV	#gene	#URDV/ gene	Ð#subjects
Cilium	63 (20.3%)	35 (17.2%)	1.80	54 (21.3%)	53 (16.1%)	33 (16.3%)	1.61	49 (19.4%)
Cytoskeleton	72 (23.2%	42 (20.6%)	1.71	61 (24.0%)	76 (23.0%)	48 (23.6%)	1.58	65 (25.8%)
ECM	46 (14.8%)	22 (10.8%)	2.09	40 (15.7%)	38 (11.5%)	16 (7.9%)	2.38	35 (13.9%)
WNT-signaling	44 (14.1%)	31 (15.2%)	1.42	41 (16.1%)	51 (15.5%)	32 (15.8%)	1.59	46 (18.3%)
Cell migration	77 (24.8%)	52 (25.5%)	1.48	70 (27.6%)	66 (20.0%)	44 (21.7%)	1.50	55 (21.8%)
Unique subtotal	185 (59.4%)	113 (55.4%)	1.63	128 (50.4%)	181 (54.8%)	110 (54.2%)	1.65	125 (49.6%)
Other ontologies	125 (40.6%)	89 (44.1%)	1.36	48 (18.9%)	150 (45.2%)	93 (45.8%)	1.61	60 (23.8%)
No URDVs	–	–	–	78 (30.7%)	–	–	–	67 (26.6%)
Total	310	202	1.56	254	331	203	1.63	252

Note. Count of URDV, or neural tube defect candidate gene, or subjects with myelomeningocele are shown with the percentage to the total count of each column in bracket. Some genes are classified to more than one category. Count of URDVs, or gene or individual are presented follow by the percentage of the count presented in bracket. Some genes can be assigned to more than one ontology categories. A unique subtotal count shows the subtotal number of the URDV, or gene, or subject without double counting when a gene has multiple ontology groups assigned. Not in above – represent URDVs, genes or subjects outside the five ontologies. URDV—ultra-rare deleterious variant, EA—European American subject, MA—Mexican American subject. ECM – extracellular matrix.

#### Subjects had multiple URDV-containing genes affecting one or multiple ontologies

Recently published articles demonstrated the genetic contribution in individuals affected with NTDs that were potentially digenic heterozygote with de novo and/or rare deleterious variants inherited from each parent^12^. In this study, nearly 30% of MMC subjects had two or more URDVs in different NTD candidate genes belonging to the same and/or different ontologies. Overall, 85 EA MMC and 91 MA MMC exomes had multiple URDV-containing genes classified with different ontologies (Supplementary Table 3). Approximately 40% of these subjects had URDV-containing NTD candidate genes representing various combinations of the five ontologies presented above.

#### Oligogenic heterozygote URDVs involving the same ontology

A subset of MMC subjects including 25 EA and 21 MA were carrying two to three URDV-containing candidate genes that belong to the same ontology group (Table 2). Nine EA MMC and four MA MMC subjects had URDVs found in two candidate genes associated with cilia structure/function (Table 2). Of note, many cilium gene products also play roles in one or more of the other four ontologies examined here. Two EA MMC and five MA MMC subjects had URDVs in two genes with functions associated with WNT signaling (Table 2). Eleven EA MMC and nine MA MMC subjects had URDVs found in two genes with functions associated with cytoskeleton structures and functions (Table 2). Three EA MMC and one MA MMC subjects had URDVs found in two or three genes with functions associated with ECM structure and functions genes (Table 2). Finally, seven EA MMC and 10 MA MMC subjects had URDVs found in two or more candidate genes regulating cell migration (Table 2). Fifteen EA MMC subjects and 20 MA MMC subjects were carrying multiple URDV-containing candidate genes in different ontological groups.

**Table 2:**
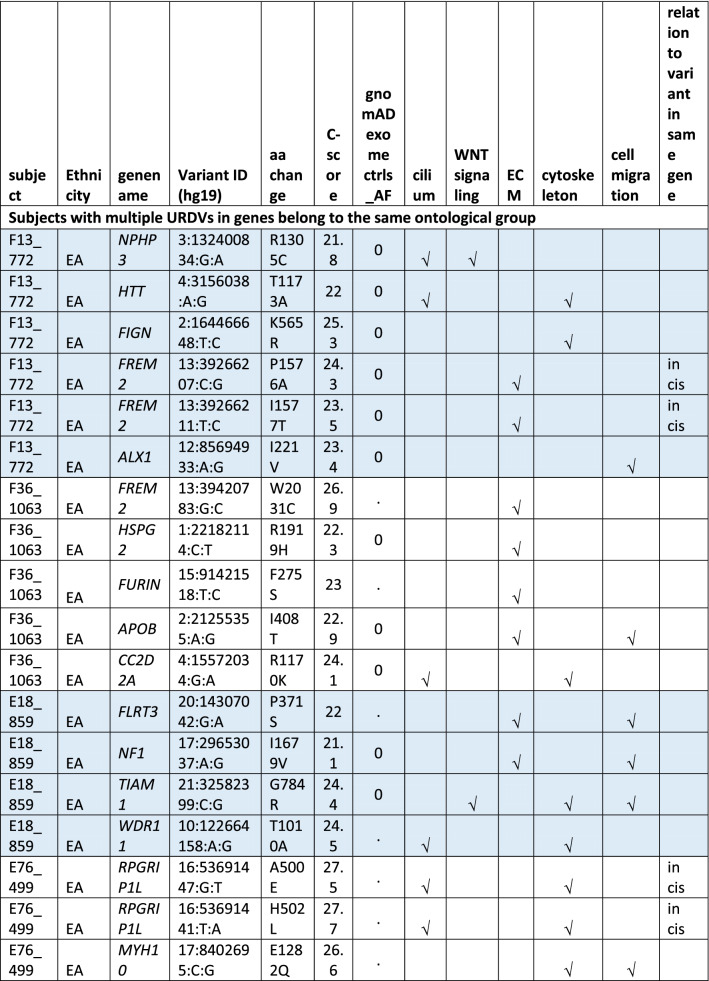

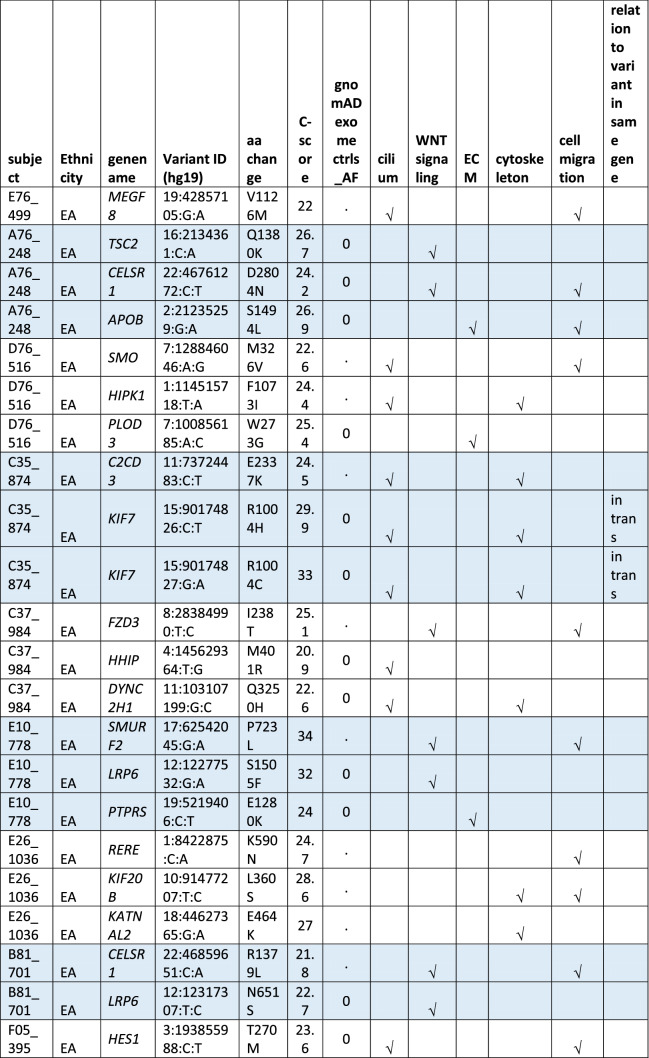

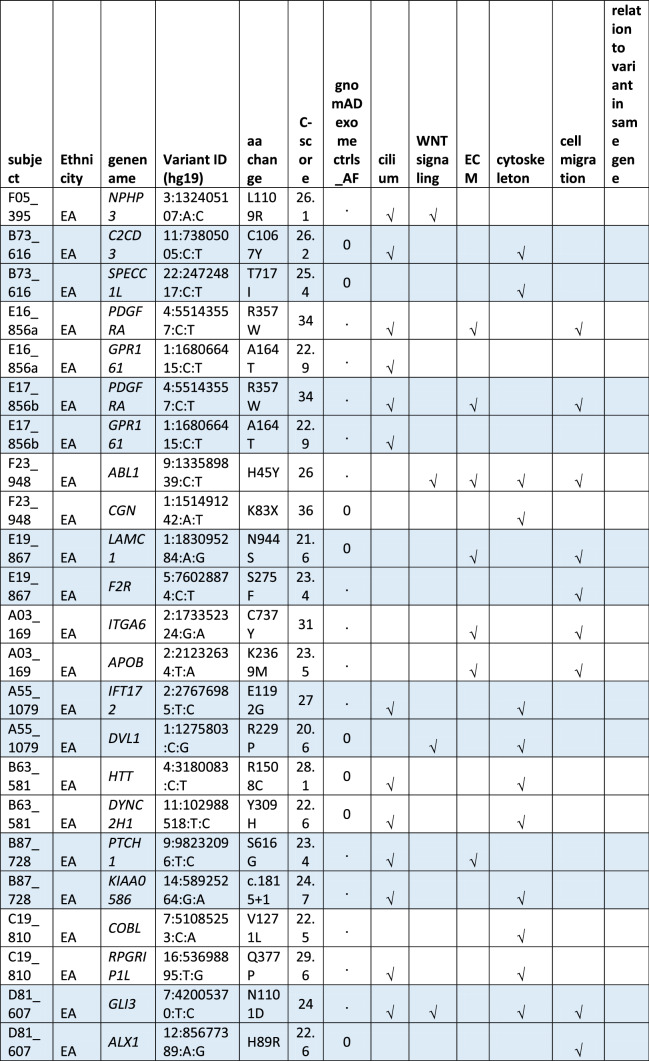

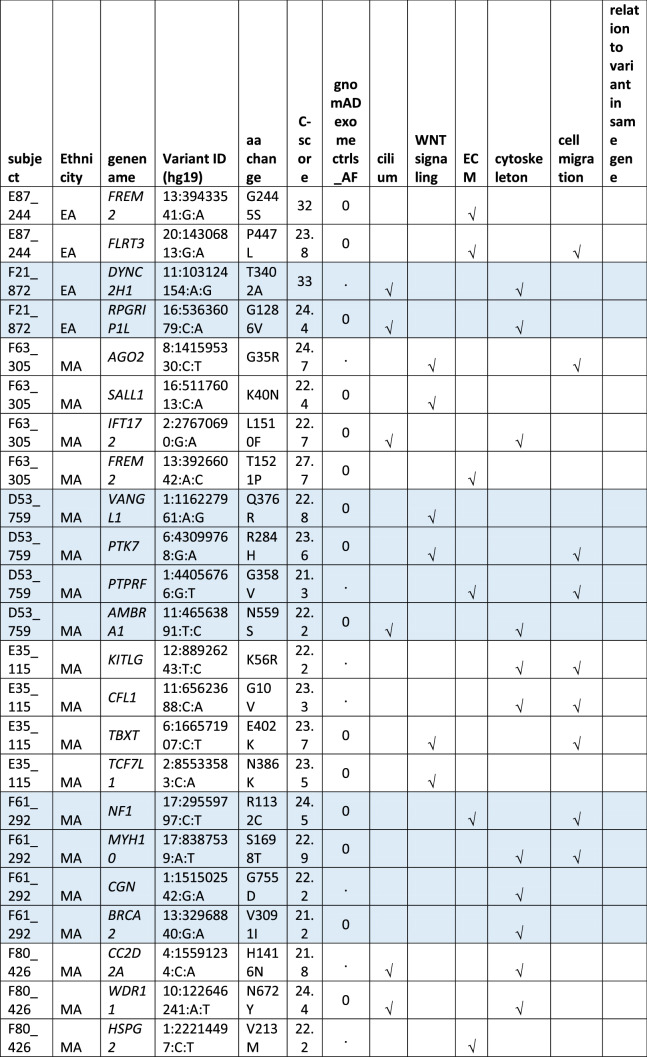

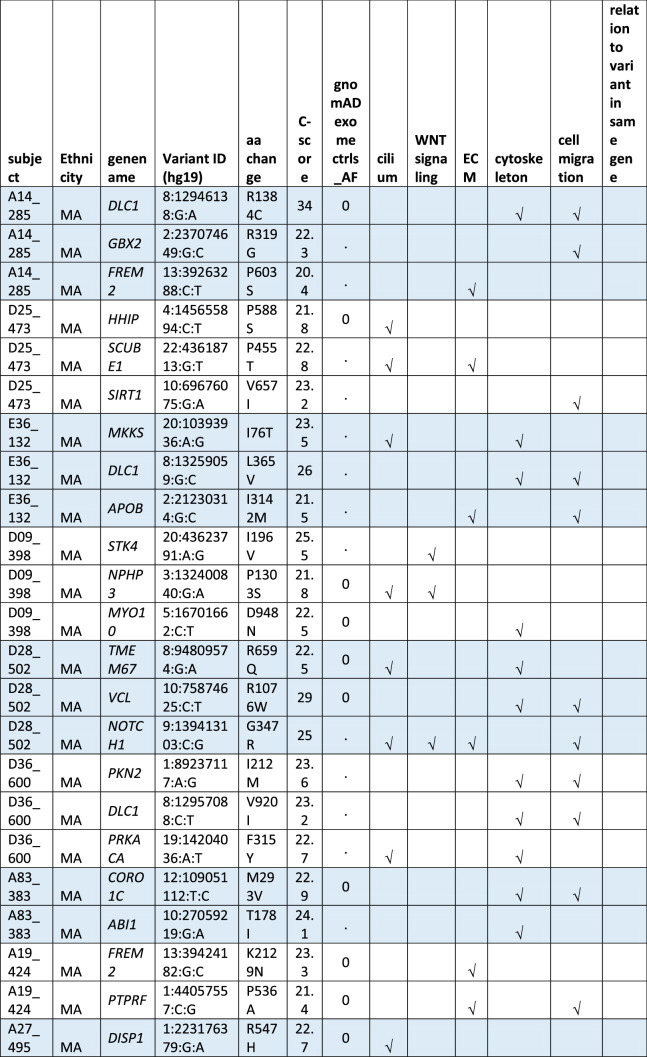

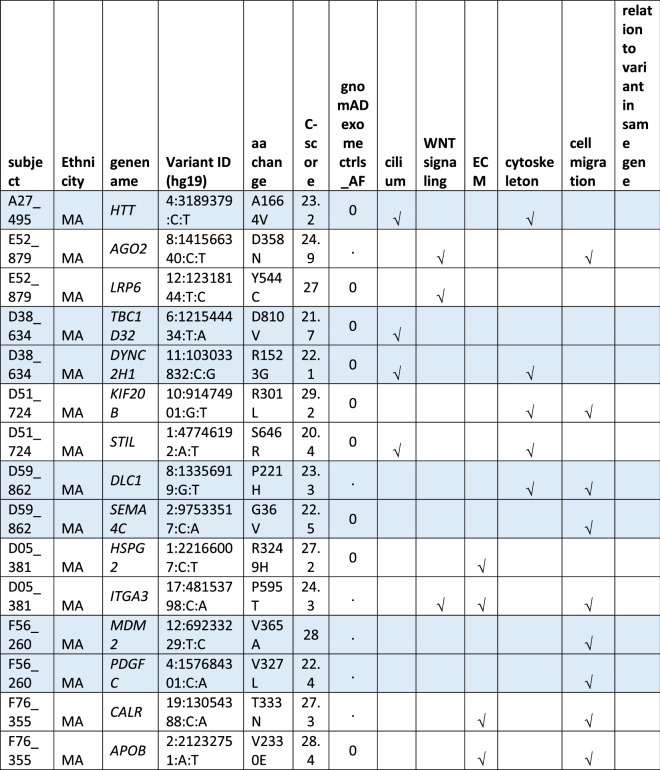
Subjects with multiple URDVs in candidate genes classified with one or more ontological groups.

Note: EA—European American subjects, MA—Mexican American subjects. URDV—ultra-rare deleterious single nucleotide variant defined in Materials and Method section. Gene name—is the symbol of gene recommended by the Human Genome Organization Gene Nomenclature Committee (HGNC). Gene assigned into cilium, WNT-signaling, extracellular matrix (ECM), cytoskeleton and cell migration was described in Materials and Methods, and Supplementary Table 3. C-Score—Combined Annotation Dependent Depletion (CADD) score is a broadly applicable metric that objectively weights and integrates diverse information that integrates multiple functional annotations into one metric by contrasting variants that survived natural selection with simulated mutations.

#### URDV-containing genes and human neural tube expression and mouse phenotypes

The expression profiles of genes for four or more human neural tubes at Carnegie Stages (CS) 12 and 13 by SAGE were available for use to annotate NTD candidate genes with URDVs^22^. Expression of nearly 40% of the 302 URDV-containing NTD candidate genes was consistently detectable in CS12 and/or CS13 human neural tubes compared to less than 15% of 249 genes with no URDV identified (Supplementary Table 2). A Chi-square test comparing NTD candidate genes with and without URDVs showed significantly more URDV-containing genes expressed in human CS12 and CS13 (p < 0.0001, Supplementary Table 2). In addition, over 80% of the 302 URDV-containing NTD candidate genes caused embryonic lethality on or before neural tube closure in knockout mice of these genes^9^.

## Discussion

This study revealed that 70% of the 506 MMC subjects consisting of the two ethnic groups with the highest prevalence of myelomeningocele in North America carry ultra-rare deleterious variants (URDVs) in 302 genes previously demonstrated to cause NTD phenotypes in animal models^9^ or associated with human NTDs (Supplementary Table 2). Ontology enrichment analyses showed around 50% of subjects in the cohort have one or more URDV-containing NTD candidate genes potentially impairing the structure and/or function of one or more ontological groups including cilium, SHH and WNT signaling, remodeling ECM and cytoskeleton, and cell migration. Normal structure and function of these genes are necessary for successful closure of the neural tube in animals^1,3^. The study results shown here may have a high impact on our understanding of the genetic mechanisms of human MMC.

One-fifth of MMC subjects in the study had URDVs in NTD candidate genes affecting cilium structure and/or function, suggesting human myelomeningocele risk is very likely associated with ciliopathy. The extent of URDV-containing ciliopathy genes associated with human NTD cases is not known. A review article by Vogel et al. (2012) noted that some human syndromes such as Meckel-Gruber syndrome and Joubert Syndrome that present with NTDs were associated with ciliopathies and recommended genetic screening of ciliopathy genes for human NTD cases^23^. Both NE and NNE involve the primary cilium playing roles in transducing extracellular signals to regulate cell growth, proliferation, directional migration and adhesion vital to neural tube development^1,2,24–29^. Many mutant mouse models with loss of function in the cilia-associated genes developed NTDs^9^. A high proportion of copy number variations in cilia genes have also been found in subjects affected by NTDs^30^. In this study, heterozygous URDVs identified in *B9D1, CC2D2A, INPP5E, KIAA0586, KIF7, MKS1, NPHP3, PIBF1, RPGRIP1L*, and *TMEM67* belong to ciliopathy genes known to contribute to autosomal recessive syndromes MKS and JBTS in humans. In addition, there were cilium dependent Hedgehog signaling genes: *DYNC2H1, GLI2, GLI3, GPR161, HHIP, IFT172, IFT57, IFT88, KIF7, MKS1, PRKACA, PTCH1, RPGRIP1L, SMO,* and *TULP3* present in MMC exomes. This study also found digenic heterozygous URDVs in two cilia genes in 13 MMC subjects. Several subjects had two URDV-containing genes classified to sub-functional groups such as Smoothened signal regulation (i.e. *C2CD3-KIF7*), Hedgehog off state (i.e. *DYNC2H1-RPGRIP1L*), Hedgehog signaling (i.e. *HHIP-DYNC2H1*), and dynein intermediate chain binding (i.e. *DYNC2H1-HTT*). It is likely that two genes within the same sub-functional group could contribute to digenic impairment of ciliary structure and/or functions critical to neural tube closure increasing the risk of MMC in these cases.

The PCP pathway modulates cellular functions such as ciliogenesis, actomyosin cytoskeleton and microtubules remodeling that are critical to neural tube formation^1,31–36^ . Normal ciliogenesis is important for non-canonical WNT PCP signaling^37^. Many studies showed defects in cilia can lead to canonical WNT signaling over-activation^29^. Furthermore, some evidence suggests cilium is a potential regulator (molecular switch) between canonical and non-canonical WNT signaling^38^. Neural tube formation involves biomechanical mechanisms resulting in mediolateral convergence and rostro-caudal extension of neural plate and axial tissues^1^. The convergent extension process depends on normal non-canonical WNT/PCP pathway activity. WNT/PCP activity influences remodeling of the cytoskeleton that drives events such as reshaping cells and bending the neural plate. Deleterious genetic variants disrupting normal function of core PCP maintenance genes (e.g. *CELSR1, DACT1, SCRIB* and *VANGL2*) has been associated with ~ 20% of craniorachischisis cases and 8% of spina bifida cases^3,39^. The extent of non-canonical WNT/PCP gene variants identified from subjects affected by various types of NTDs was recently published^19^. This study identified 7% of total MMC subjects with one or more URDVs affecting the WNT/PCP signaling pathway and 3–4% affecting core PCP pathway genes involved in neural tube closure. A handful of WNT signaling genes were shown to have higher mutational burden in the current study cohort^40^. Two URDVs [i.e. *VANGL1* p.(V239I) and *LRP6* p.(Y544C)] in two Mexican American subjects in the study cohort had been shown to cause functional loss of the proteins^41,42^. Discovery of heterozygous digenic deleterious variants in human NTD cases with spina bifida (i.e. *PTK7/ SCRIB*) and anencephaly (i.e. *CELSR1/SCRIB; CELSR1/DVL3*) strongly support the strategy of screening PCP genes to discover risk alleles contributing to human NTDs^3,12^. Here, we identified seven new digenic combinations of WNT/PCP signaling genes with three of these combinations (i.e. *CELSR1-LRP6*, *SMURF2-LRP6*, and *PTK7-VANGL1*) involving the core PCP pathway. Of these, three subjects had URDVs potentially interfering with regulation of planar polarity establishment (GO:0090175). These new gene combinations suggest previously unknown gene–gene interaction candidates for validation with animal studies in the future.

Knowledge of the extent of genetic risk of cytoskeleton structure function components to human MMC is limited. A quarter of the study subjects have one or more URDVs in 65 NTD candidate genes coding for cytoskeleton components to make microtubule structures: actomyosin filaments forming cell projections such as filopodia, lamellipodia, axoneme and cilia. Myosin motors along actin filaments facilitate vesicular cargo and biomolecule transport throughout the cell in response to the cues of signaling molecules transmitted from cilia^37^. In a study of 43 NTD trios, Lemay et al. (2015)^43^ identified heterozygote de novo deleterious variants in the *SHROOM3* gene of one MMC case and one anencephaly case. Only one MMC subject in this study had a *SHROOM3* URDV. Interestingly, half of the 65 cytoskeleton associated genes in MA and two-third in EA are associated with cytoskeleton and cilium, and the remaining genes belonging to actin cytoskeleton only. Digenic heterozygous URDV combinations found in MMC subject included *C2CD3/KIF7*, *CC2D2A/WD11*, *DYNC2H1/RPGRIP1L* and *HHT/DYNC2H1* affecting cytoskeleton and cilium; and *ABL1/CGN*, *KIF20B/KATNAL2* and *MYH10/CGN* affecting actin cytoskeleton function.

Nearly 15% of the subjects in this study had URDVs in NTD candidate genes coding for ECM components. The ECM compartment serves as a microenvironment retaining growth factors and signaling molecules (e.g. SHH, BMP, WNT, EGFs and FGFs) to recruit or influence neuroepithelial cells to determine shape, size, polarity and migration status for neural tube closing^28^. The roles of ECM components on regulating the position of the nucleus, cell shape, proliferation, migration, and morphogenesis of neural tube has been well-discussed^1^. Loss of function in some ECM components (e.g. *Frem2 and Hspg2*) caused NTDs in animal models^9^. This study is the first to reveal URDVs in *FREM2*, which were present in around 3% of study subjects, and URDVs in *HSPG2*, which were present in 2% of study subjects. Pathogenic variants in *FREM2* have been associated with Cryptophthalmos (#123,570) and Fraser syndrome 2 (#617,666) with skull abnormalities or encephaloceles^44^. Digenic heterozygotes in two to four ECM genes were present in eight subjects in this study.

URDVs in cell migration genes were present in over 20% of the study subjects with many of these genes also involved in cilia, cytoskeleton, ECM, and WNT signaling. Ventral-dorsal migration of cells into the neural fold and rapid cell proliferation contribute to bending of the dorsolateral spinal neural plate, facilitating neural tube closure^45^. The *ct* mouse mutant has increased hindgut cell proliferation due to abnormal *Grhl3* expression at the caudal region which surpasses NE and NNE migration and proliferation and prevents spinal neural tube closure^5,46^. Knockdown of the cell migration molecule *Itgb1* prevents closure of the neuropore^47^. Mice with ablation of *Rac1*, a molecule modulating actin cytoskeleton remodeling and cell migration in NNE, developed open spina bifida, exencephaly or anencephaly^48^. In addition, impaired mesodermal cell migration due to diabetic pregnancy led to NTDs in mouse^49^. Seventeen MMC subjects in this study had oligogenic heterozygous URDVs in genes involving in cell migration providing examples for testing oligogenic heterozygote effects on cell migration as a mechanism of MMC development.

**Figure 6:**
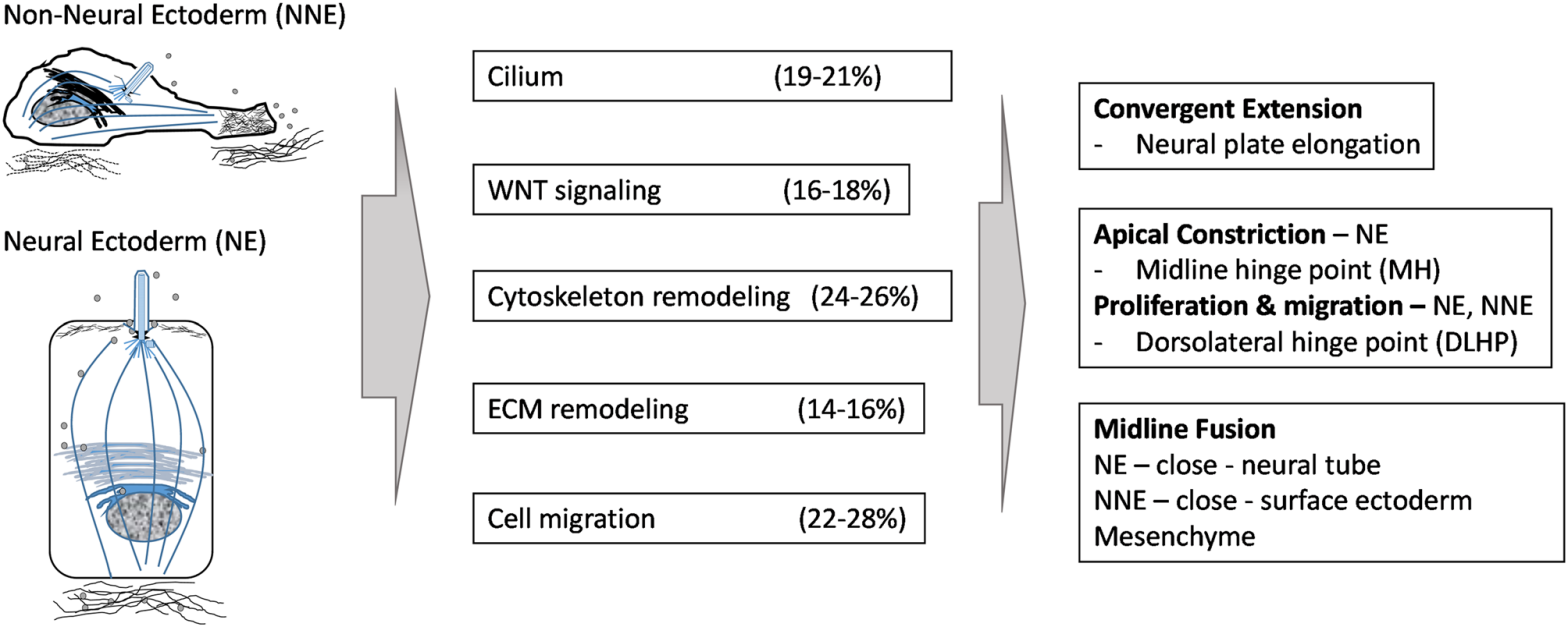
Summary of proportion of study subjects with URDV-containing NTD candidate genes constituting the components of cilium structure and function, WNT-signaling, cytoskeleton remodeling, extracellular matrix (ECM) remodeling and cell migration. URDVs were identified in the DNAs extracted from blood lymphocytes and expected to be present in all body cell types including both neural and non-neural ectodermal cells (NE and NNE respectively). Identifying the URDVs is the first of many steps leading to the understanding of the genetic mechanisms of human MMC development. Cross interaction of genes within and between the five groups is anticipated with many genes assigned to more than one of these groups. Presence of multiple genes with URDVs in one subject provide basis for testing potential gene–gene interaction that may interrupt cell proliferation, or convergent-extension, or apical constriction, or midline fusion or a combination of these processes leading to MMC.

Knowledge of oligogenic gene–gene interaction between genes of different ontologies contributing to risk of myelomeningocele is lacking. Oligogenic inheritance in holoprosencephaly, a rare birth defect of the brain, was recently demonstrated^18^. Evidence of mouse embryos with oligogenic heterozygous PCP related genes knock-out developed spina bifida suggesting a subject having heterozygous URDVs in two NTD candidate genes belonging to the same ontology group may increase risk of MMC development^9,17^. So far, no non-syndromic human spina bifida cases with homozygous deleterious variants in one gene has been found, and only cases with digenic heterozygous rare deleterious variants in two PCP genes had been reported^12,13^. This study cohort showed examples of MMC subjects carrying multiple NTD candidate genes containing URDVs and one subject has two compound heterozygous URDVs in the *KIF7* gene. Figure 6 summarized the potential impact of URDVs in NTD candidate genes identified from 506 MMC subjects. Hypothesis-free testing of random combinations of gene–gene interaction in animal models is too labor intensive and cost-prohibitive. While MMC risk for some subjects might be rationalized by disrupted genetic interaction between URDV-containing genes within the same ontology, subjects with URDV disrupted genes in different ontologies will be challenging. Here, the study results provide examples of new highly probable genetic interacting partners elevating MMC risk through an oligogenic heterozygote mechanism. For example, subject E52-879 has URDVs p.(D358N) in *AGO2* and p.(Y544C) in *LRP6*. While interaction of *AGO2* and *LRP6* has not yet been demonstrated, it has been shown that inactivated canonical WNT signaling facilitated nuclear entry of miR-133a and complexed with AGO2 to suppress *DnmT3b* expression in mouse HL-1 cells^50^. Also, the presence of p.Y544C impaired LRP6 localization to plasma membrane for facilitating WNT signaling^42^. Subject D22-469 carries URDVs p.(N35K) and p.(H31R) respectively in *SNX3* and *CITED2*, known components for WNT signaling and cell migration respectively. SNX3 binds WLS to regulate recycling of WLS thereby regulating the level of WNT excretion. Furthermore, it has been demonstrated that SNX3 with the subject’s URDV p.(N35K) failed to interact with WLS affecting WNT sorting and secretion^51^. A recent report demonstrated the developmental defects of *Cited2* morphants in Zebrafish could be rescued with exogenous WNT5A and WNT11^52^.

This study identified URDVs in NTD candidate genes in 70% of MMC subjects lending strong support to the approach of screening NTD candidate genes to identify high impact genetic risk factors associated with human MMC development. Results of oligogenic URDV-containing cases present in this study provide a rational basis to test oligo-genic combinations in animal models to elucidate the mechanism(s) of MMC in humans. New oligogenic disease mechanism(s) for MMC development can be discovered from the new combinations of genes presented in this study and not previously known to cause NTDs. Testing genetic interactions between URDV-containing NTD candidate genes discovered from MMC subjects to understand human MMC mechanism is sound and promising.

In the study, we observed the presence of URDV-containing candidate genes that were common to EA and MA, and unique to one or the other ethnic population. Many of the genes unique to EA or MA belongs to the same ontology subclasses and a small number of genes belong to distinct specific subclasses. Differences in subclass information may help to reveal the disease mechanisms for the subjects who carried these variants. The potential of utilizing the observed differences in these genetic variants to predict differential risks in different ethnic populations may require replication with additional studies.

The study results were limited to examining the impact of ultra-rare SNVs in the coding region and the splice sites of NTD candidate genes. The impact of URDVs in genes not known to cause NTDs in mice has not been evaluated. Yet there are a significant portion of genes which have important biological function and are detected in the developmental stages when the neural tube closes that can affect neural tube development. Importance of rare deleterious variants, out of frame indel variants, deleterious non-coding variants and copy number variants should be recognized. Previous genetic association studies demonstrated some impact of common and rare deleterious variants to NTD risk in humans but generally lack replication due to various limitations of association study designs, ethnic background, and sample size. This study cohort has similar limitations. Separate analysis using all the common variants identified from the subject exomes did not yield significant association with the selected NTD candidate genes after correcting for multiple testing.

## Materials and methods

### Study population

A total of 511 subjects were selected for whole exome sequencing (WES) from a myelomeningocele study cohort enrolled from spina bifida clinics in five locations of North America between 1997 and 2010 (Au et al., 2008)^53^. All subjects provided an informed consent and enrolled in accordance with an institutional IRB at the University of Texas Health Science Center (UTHealth) at Houston. WES was performed on DNAs of 257 EA MMC including 140 females and 117 males, and DNAs of 254 MA MMC including 134 females and 120 males. Subjects in the study are sporadic cases and reported no family history of MMC. The majority (81%) of the study subjects were born before January 1998, the date North American countries mandated fortification of food crops with folic acid^54^. Fifty-two subjects were born in 1998 and 46 were born after 1998. Approximately 24% of the study subjects have MMC lesions at or above vertebrae L1 up to T10, 66.7% at or below vertebrae L2 down to sacrum, 1.7% had lesion spanning L4 to T10, and 8.2% had no specific lesion level information. Around 60% of the study subjects had hydrocephaly, 5.5% had arrested hydrocephaly, 3.7% had no hydrocephaly and 31.1% had no specific information. The majority subjects (74.8%) did not have information on Chiari Malformation, 0.6% had type I and 22.5% had type I Chiari Malformation, and 2.2% were normal. Blood samples were collected from subjects and parents where possible and genomic DNA was extracted for the study.

### Exome sequencing and variant annotation

Exome library probes were made from an in-house design based on TargetSeq (Life Technologies, Inc.) with addition of splice sites, UTRs, small non-coding RNAs (e.g., microRNAs) and a selection of miRNA binding sites, and 200 bp promoter regions. High quality genomic DNA samples were processed using the exome library probes and the captured DNA products were sequenced following the manufacturer’s standard protocol for multiplexed sequencing using the P1 chip on the Ion Proton platform (Life Technologies, Inc.). Quality of sequencing was maintained at 40–60 million reads with read length between 120 and 150 bases, and over 75% reads were on-target for all successfully sequenced samples. Other quality control measures were implemented to map 45–60,000 SNP per sample with ~ 50% heterozygote variants and the Ts/Tv ratio average of 2.5. Samples that failed to meet the above quality criteria were re-sequenced. If re-sequencing failed, another subject’s DNA will be used to reach the goal of sequencing at least 500 subjects.

For sequence data that passed variant level and sample level quality filters, variant calling was conducted using Genome Analysis Toolkit GATK HaplotypeCaller version 3.x following best practice guidelines^55^. Additional variant filtering steps had been described previously^21,40^. Briefly, only variants designated a “PASS” by Variant Quality Score Recalibration (VQSR), met map quality score < 20, and having inbreeding coefficient < -0.3 were retained for further analysis. Individual sample filters were used to ensure that only high-fidelity variants with alternate allele depth > 25%, a read depth > 10, and genotype quality score > 20 were analyzed. Allele count (AC), allele number (AN), and allele frequency (AF) were recalculated for individual ethnicities after the filtering processes. Filtered high quality single-nucleotide variants (SNVs) were annotated using the non-synonymous single-nucleotide variant functional predictions (dbNSFP)^56^ version 4.0 database for all functional prediction information publicly available. Further analysis was focused on SNVs leading to stop_gained, stop_lost, non-synonymous, splice donor, and splice acceptor site changes in the canonical transcripts.

Thirty-three EA subjects and 55 MA subjects in the study were also re-sequenced using Illumina NGS platform (i.e. NovaSeq). Raw sequence reads of WES were mapped to GRCh38 (hg38) and read for WGS were mapped to GRCh37 (hg19). Variants were called following the GATK best practice guidelines. Then the variant data generated was filtered using the same filtering criteria described above for the Ion Proton generated data. Filtered variant data of the 88 subjects generated by the Ion Proton platform used in the study were extracted and compared to filtered variant data generated by the Illumina Platform to verify variants with concordance.

### Ultra-rare functional deleterious SNVs (URDVs) analysis

The alternate allele frequencies (aAFs) of variants reported for the exomes of the non-Finnish European (NFE) and Ad Mixed American (AMR) control populations in the genome aggregation database gnomAD v.2.1.1 (https://gnomad.broadinstitute.org/) were used to annotate the single nucleotide variants (SNVs) in the study^57^. Variants having ethnic aAF = 0 or absent in gnomAD v2.1.1 (controls) despite high location coverage in gnomAD were defined as ultra-rare SNVs (URVs). Variants having aAFs in NFE or AMR gnomAD v2.1.1 (controls) less than 0.01 were defined as rare, whereas aAF ≥ 0.01 were defined as common. Combined Annotation Dependent Depletion^16^ (CADD; https://cadd.gs.washington.edu/) phred scores (C-score) of variants were used as the model to predict deleteriousness and variants with a C-score ≥ 20 (top 1% most deleterious) cutoff were defined as ultra-rare deleterious variants (URDVs) and retained for further comparison in the study. Genes with URDVs were further evaluated for potential association with MMC in the study cohort using the in silico function analysis tools. In addition, all URDVs identified in the NTD candidate genes in Supplementary Table 3 had been manually examined and confirmed using IGV Web App (https://igv.org/app/)^58^.

### Online variant analysis tools

Annotation of gene and function with Gene Ontology^59^ (GO; http://geneontology.org) terms were used to estimate the extent of enrichment for particular ontology in molecular function, biological process, cell components and pathways in each group of genes found with URDVs to evaluate their potential relevance to development of neural tube defects. Several web based tools were used including PubMed and Mouse Genome Database^9^ ( http://www.informatics.jax.org/phenotypes.shtml) to compile a list of genes known to associate with neural tube defects (Supplementary Table 2) searching for terms: neural tube defects, open neural tube, spina bifida, anencephaly, exencephaly, craniorachischisis, and abnormal neural tube morphology. ToppFun (https://toppgene.cchmc.org/enrichment.jsp) and ToppCluster^20^ (https://toppcluster.cchmc.org/) were used to evaluate ontology enrichment and for comparing multiple groups of genes (Chen et al. 2009). Genes in the GO terms were extracted using g:Profiler^60^ (https://biit.cs.ut.ee/gprofiler/convert). Gene tolerant to loss-of-function mutation (pLi score) and haploinsufficiency were annotated using resources from DatabasE of genomiC varIation and Phenotype in Humans using Ensembl Resources(DECIPHER; http://decipher.sanger.ac.uk)^61^.

To annotate expression of the genes of interest in animal neural tube, a database for human neural tubes during Carnegie stages CS12 and CS13^22^ (Krupp et al., 2008) was used. Genes expressed in mouse future spinal cord between Theiler Stages TS11-19 and early embryonic lethality mouse genes (MP:0008762) were extracted from MGI (http://www.informatics.jax.org/) for function annotation.

### NTD candidate genes

Increasing numbers of published reports showed compound heterozygote deleterious variants of mouse NTD genes in human NTD subjects suggesting these variants as genetic risk factors for human NTDs. To facilitate variant containing gene prioritization, a list of 551 genes (Supplementary Table 2) was assembled from Mouse Genome Database^9^ (http://www.informatics.jax.org/) using search terms: open neural tube (MP:00000929), exencephaly (MP:0000914), anencephaly (MP:0001890), craniorachischisis (MP:0008784), and abnormal neural tube closure (MP:0003720). Also included were *RARA*, *RARG*, *KIAA0586* an*d KATNAL2* with evidence of open neural tube defects in mice, chicken, frog and human^62–64^. Mouse NTD mutants not assigned to a gene and genes assigned only to spina bifida occulta were not included in this study. In addition, a list of 14 genes were added because association has been established in our study cohort (Supplementary Table 2). Together, a total of 551 candidate genes were selected for the study.

### Ethical compliance

Affected subjects and/or their parents were recruited for the research study with a consenting protocol approved by the University of Texas Health Science Center (UTHealth) at Houston Institutional Review Board.

## Supplementary Information

Supplementary Information.

## Data Availability

Variants information is present in Supplementary Table 3. Also, variants will be available for review upon publication at dbSNP (https://www.ncbi.nlm.nih.gov/snp/) under BioProject ID: PRJNA611755.
